# Fear of Deportation and Hispanic Early Adolescent Substance Use: a Moderated Mediation Model of Stress and Hope

**DOI:** 10.1007/s11121-023-01593-3

**Published:** 2023-11-17

**Authors:** Ronald B. Cox, Hua Lin, Robert E. Larzelere, Juan Bao

**Affiliations:** 1 Institute for Public Health Practice, Research and Policy, College of Public Health, University of Iowa, Iowa City, IA 52242 USA; 2 Public Policy Center, University of Iowa, Iowa City, USA

**Keywords:** Hispanic early adolescents, Fear of deportation, Stress, Substance use, Hope

## Abstract

Reports of deportation can create a state of chronic fear in children living in mixed-status immigrant families over their own or a loved one’s potential deportation. One indicator of health disparities among youth is elevated rates of alcohol, tobacco, and other drug use (ATOD). Yet little is known about the effects of fear of deportation (FOD) on ATOD or what might promote resilience. We explore the associations between FOD and ATOD use, how stress mediates this relationship, and whether hope moderates the mediated pathway from FOD to ATOD. Participants were 200 first- and second-generation 7th grade Hispanic youth (49% female) assessed across three waves of data. A moderated mediation model tested the indirect effect of FOD on ATOD through stress and whether hope moderated these associations. FOD was measured by the Family Fear of Deportation Scale. Snyder’s Children’s Hope Scale measured hope. Stress was measured by a short version of Pediatric Psychological Stress Measure. ATOD was adapted from the Monitoring the Future project. FOD was not directly associated with ATOD use. However, this path was fully mediated by stress. Hope significantly moderated the path from FOD to stress such that a one unit increase in hope completely offset the effects of FOD on stress. Hope did not moderate the path from stress to ATOD use. Interventions that increase awareness of deportation trauma, alleviate stress, and promote hope may help prevent, delay initiation into, and/or decrease ATOD among Hispanic first- and second-generation youth.

## Introduction

The consequences of underage alcohol, tobacco, and other drug use (ATOD) are a major public health concern in the USA. There are more deaths, illness, and disabilities in the USA from ATOD than from any other preventable health condition (NIDA., 2020). Adolescence is a critical risk period for the initiation of ATODs with use during adolescence signaling future deficiencies in social functioning and physical and mental health (McGue & Iacono, 2005), as well as substance use disorders in adulthood (King & Chassin, 2007). For instance, 15.2% of people who began drinking by age 14 eventually developed alcohol abuse or dependence compared to 2.1% of those who began drinking at age 21 or older (SAMHSA, 2013).

Early initiation of ATOD among Hispanic immigrant youth is particularly troubling for the nation’s public health. On average, Hispanics have an elevated rate of early initiation of substance use relative to Black and non-Hispanic White youth (Miech & Patrick, 2020). According to the 2019 National Youth Risk Behavior Surveillance System survey results (Centers for Disease Control & Prevention, 2020), about one in five Hispanic adolescents (18.4%) reported drinking alcohol and one in ten (9.5%) had tried a cigarette before age 13 years. Innovative solutions to prevent or delay ATOD initiation are needed to reduce health disparities for this population.

The prevalence of psychosocial stress among adolescents in the USA is also of growing concern. The American Psychological Association’s Stress in America Survey (American Psychological Association, 2012) indicated that adolescents report higher levels of stress than adults and that approximately one-third of adolescents feel overwhelmed (31%), depressed (30%), or tired (36%) due to stress. Others have found that psychosocial stress is associated with an escalation of adolescent drug use even after controlling for age and peers (Hoffman et al., 2000). In addition to the stressors and daily hassles common to all adolescents (e.g., new romantic relationships and changing body appearance) and the stressors common to minorities of color (e.g., poverty, racism), many Hispanic first- and second-generation immigrant youth also face stress related to the process of acculturating to the customs, values, and beliefs of a new host society in the case of first-generation youth, learning to navigate the demands of two cultures (i.e., the US culture and their parents’ culture) as is the case for second-generation youth, and how to manage family relationships when parents and children increasingly have difficulties communicating in a shared common language (aka. Shared Language Erosion; Cox, deSouza, et al., 2021) as occurs in first- and second-generation youth. Unfortunately, this also may include managing the fear that one of their family members, friends, or they themselves might be deported. Although a growing literature examines the effects of acculturation on the health and well-being of Hispanic youth (e.g., Telzer, 2010), little is known about the effects of FOD on ATOD and what factors might moderate its effect.

Despite exposure to numerous environmental barriers, most Hispanic immigrant youth demonstrate extraordinary resilience and develop into well-functioning adults (Suárez-Orozco & Suárez-Orozco, 2009). Many researchers have identified this phenomenon as the “immigrant paradox” referring to findings that immigrants progress as well as or better than their US-born peers in terms of academic and health outcomes (Coll & Marks, 2012). When immigrant families first arrive to the USA, they tend to be very hopeful of their opportunities and future. Even when facing stress-inducing obstacles and prejudice in the new host country, they often view their plight as more favorable relative to the circumstances that compelled them to migrate (Suárez-Orozco & Suárez-Orozco, 2009). Thus, the research findings summarized in the immigrant paradox point to the need to examine both risk (e.g., fear of deportation) and protective factors (e.g., hope) and how they may interact to promote positive youth development.

## Background

Immigration policies and politics have influenced immigrant families in the USA for decades. More recently, however, the immigration policies in the USA have undergone significant changes such as the criminalization of immigration, which occurred after the September 11th, 2001, terrorist attacks. Policies have ranged from the “Family Fairness” humane deportation policy promoted in the 1980s (Meierotto et al., 2020) to a strict “zero tolerance” policy initiated in 2018 (Polk et al., 2019). More recently, policies have shifted from an emphasis on the removal of criminal offenders located close to the US-Mexico border to raids that target non-criminal individuals throughout the country (Meierotto et al., 2020). Increased fear of immigrants and concerns regarding the rising costs of meeting the social and educational needs of immigrants has led to deportation being used as a form of immigration control in the USA (De Genova, 2013). Deportations reached record highs under the Obama administration (432,281 in 2013) and remained elevated under the Trump administration (i.e., 337,287 in 2018; Gramlich, 2020). Aggressive public policies, coupled with negative public opinion regarding immigration, have led to widespread fear among Hispanic families and communities (Meierotto et al., 2020). The traumatic impact of being separated or the threat of being separated due to deportation of parents and children has been associated with anxiety, depression, and multiple behavior disorders (Mills, 2015). A growing body of evidence shows that the children of unauthorized immigrant parents report persistent anxiety in response to the continual fear that a family member may be deported (Yoshikawa, 2011). For example, approximately 75% of foreign-born Hispanic youth report worrying some or all the time that someone they know will be deported, and 68% report knowing someone who has been deported (Gonzales et al., 2014). This sense of unremitting vulnerability increases psychological distress which consequently influences the attitudes and behaviors of the developing child/adolescent. The uncertainty associated with the threat of deportation decreases personal control over one’s environment, which in turn contributes to feelings of stress and anxiety and makes coping more challenging (Cai et al., 2020).

Despite little research examining the relationship between FOD and substance use among Hispanics, emerging studies suggest a significant association. For instance, one study found that US-citizen Hispanic adults who knew a detained or deported migrant had significant higher odds of reporting hazardous drinking or drug use disorders when compared to non-Hispanic White US citizens (Pinedo, 2020b). Similarly, adults who had a family member who was deported reported significantly higher odds of past-year prescription drug misuse compared to non-Hispanic Whites and Hispanics who did not personally know anyone who was deported (Pinedo, 2020a). Although no prior studies of which we are aware have directly examined the link between perceived FOD and substance use among Hispanic adolescents, a growing body of work has documented the associations between FOD and outcomes such as stigma (Dreby, 2015), discrimination (Toyokawa & Toyokawa, 2019), negative mental and behavioral health (Rojas-Flores et al., 2017; Yoshikawa, 2011), low parent involvement at school (Cross et al., 2019), and family economic insecurity (Dreby, 2015), all of which are potential predictors of Hispanic adolescent substance use. Higher levels of stress have been associated with increased odds of past-month drug and lifetime drug use for Hispanic adolescents (Schinke et al., 2016), and Hispanic children of detained or deported parents reported higher levels of psychological distress than those whose parents were permanent residents or had no contact with Immigration and Customs Enforcement (Rojas-Flores et al., 2017). Hispanic children with high levels of psychological distress have been found to use alcohol more frequently in comparison to other drinking subgroups, particularly among second-generation Hispanic children (Alva, 1995). Together, the growing body of research suggests that FOD may be associated with adolescent ATOD through the mediating mechanism of perceived psychosocial stress.

## Hope and Adolescent Resilience

Historically, hope has referred to a belief that good things will happen in the future (Snyder et al., 1991). More recently however, several more comprehensive and standardized goal-directed theories of hope have gained influence in the literature. Primary among them is Snyder’s cognitive, multidimensional theory of hope, which is a resiliency promoting factor that integrates three components of positive expectancies: goals, pathways, and personal agency (Snyder et al., 1997). Snyder defines hope as a “cognitive set involving the beliefs in one’s capabilities to produce workable routes to goals (the pathways component), as well as the self-related beliefs about initiating and sustaining movement toward those goals (the agency component)” (Snyder, et al., 1997, p. 401). According to the hope model, when these two types of thinking (i.e., pathways and agency) are activated to reach a goal, a high-hope person has a positive emotional mindset that sustains attention and motivation toward a task (Snyder, 2002). In contrast, a low-hope person has a negative emotional mindset, which leads to off-task thinking. Additionally, people with high hope view stressors as challenges and may successfully utilize alternate pathways in the context of stress. In contrast, people with low hope are particularly susceptible to stressors and may stop goal pursuits in the context of stress. Thus, from Snyder’s perspective, hope is a dynamic cognitive process of goal appraisal, motivation to make alternative pathways toward goal realization, and the belief that goals are attainable. The tenants are dynamic in nature in that they are reciprocal, reinforcing one another through situational experience (Gum & Snyder, 2002). Therefore, when a goal is unobtainable, high-hoping individuals will modify their goal to a more perceivably realistic goal, whereas low-hoping individuals may slip into depression and despair leading to maladaptive coping strategies (Gum & Snyder, 2002). Finally, Gum & Snyder (2002) conceptualizes goals as either positive or negative. Positive goals focus on improving one’s situation while negative goals focus on preventing or avoiding an unwanted situation. For instance, an initial goal may be positive such as finding a cure for a terminal illness. However, when a cure is unobtainable, high-hoping individuals may transition goals to symptom management (an alternative positive goal) and the reduction of distress associated with death (an adaptive negative goal) (Hoffmann, 2005).

Evidence points to positive associations between hope and adaptive functioning such as higher levels of positive affect, self-esteem, social support, life satisfaction, and self-concept among children and adolescents (Vacek et al., 2010). Higher hope levels are also associated with lower levels of suicidal ideation (Kwok & Gu, 2019) and substance use (Wilson et al., 2005). However, findings regarding the moderating effect of hope in adolescent stress and substance use have been mixed. For example, Fite et al., (2014) found significant moderating effects of hope between delinquency and 30-day frequency of tobacco and marijuana use, depressive symptoms, and frequency of alcohol use, but not between peer substance use and frequency of substance use among Hispanic high school students. Others found that stress and hope were significantly associated with negative affect, but that hope did not moderate the relation between stress and negative affect (Vacek et al., 2010). However, this study may have been underpowered to detect moderation. In another study, Wilson et al., (2005) failed to find the moderating role of hope in the relation between neighborhood social disorder and substance use among an ethnically diverse group of middle school students. However, neighborhood social disorder and lower sense of hope were significantly associated with substance use. Given the limited and divergent findings regarding the associations between FOD, stress, substance use, and hope among Hispanic adolescents, questions remain regarding how FOD may influence Hispanic early adolescents’ substance use, and whether hope serves as a moderator to mitigate the proposed indirect effect of FOD on substance use through stress.

Understanding the relationship between hope and ATOD may prove important because hope is a malleable factor that is responsive to interventions designed for adolescents (Sin & Lyubomirsky, 2009). For example, Marques et al., (2009) conducted a brief intervention to enhance hope among middle school students that resulted in significant increases in psychological strengths at an 18-month follow-up survey. Kirschman et al., (2010) found that urban adolescents’ participation in a 6-week summer camp devoted to developing dance and psychosocial competence skills was associated with increases in hope in a 4-month follow-up assessment. Findings from the Penn Optimism Program suggest that interventions aimed at increasing positive thinking can prevent mood disorders among youth (Gillham et al., 2001). Together, these studies suggest that youth who have higher levels of hope are more likely to attempt to manage life’s adverse events and are often successful. This success, in turn, leads to increased self-efficacy and positive coping. On the other hand, youth who have low levels of hope tend to give up more easily when faced with stress inducing challenges (Baxter et al., 2017). If hope is shown to moderate the path from FOD to ATOD through stress, it would suggest that hope is an important intervention target to prevent the initiation of ATOD among Hispanic immigrant youth, without having to focus on aspects of ATOD. This last point is particularly salient given the reticence of Hispanic immigrant families to attend prevention programs for adolescent ATOD (Cox, Washburn, et al., 2021).

## The Present Study

**Fig. 1:**
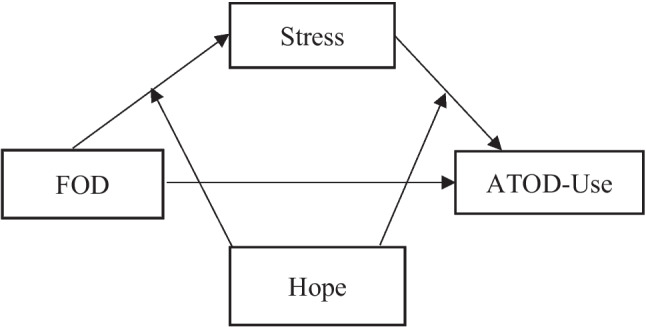
Conceptual model. Note: *β*, standardized coefficient; OR, odds ratio, ^*^p < .05, ^**^p < .01, ^***^p < .001

Guided by Snyder’s hope theory and prior literature, we aim to address the following research questions: (1) Is FOD associated with ATOD use; (2) does stress partially or fully mediate the path from FOD to ATOD; (3) does hope moderate the path from FOD to stress and, or the path from stress to ATOD, in a sample of Hispanic seventh graders (see Fig. 1, conceptual model). From these questions, we will test three hypotheses: (1) FOD is positively associated with Hispanic early adolescent ATOD use; (2) stress fully mediates the association between FOD and Hispanic early adolescent ATOD use; and (3) the indirect effect of stress on FOD and ATOD only exists when the Hispanic early adolescents have lower levels of hope, such that hope moderates the path from FOD to stress and the path from stress to ATOD. We also include child gender as a covariate to control for the influence of traditional gender roles on ATOD among Hispanic youth (Wahl & Eitle, 2010).

## Methods

### Participants

The current study used three waves of data (i.e., baseline in January or February, 4 months post-baseline in May, and 10 months post-baseline in November or December; referred to respectively as Time 1, Time 2, and Time 3) from the *¡Unidos Se Puede!* project, a family-based prevention intervention designed to increase academic achievement among Hispanic immigrant youth (Cox, 2017). Participants were 200 Hispanic first- and second-generation immigrant youth (49% female) attending the seventh grade at Time 1 data collection (youth were in the 8th grade for Time 3 data collection) in an urban school district in the Southern United States. Most youth (55%) reported being born in the USA, 18% preferred to take the Spanish version of the surveys, 81% reported being of Mexican descent, and 73% reported that both parents had a high school education or less. Approximately 95% of the sample reported receiving free or reduced lunch at school.

### Measures

#### Past 30-Day Youth ATOD Use

Youth ATOD was adapted from the Monitoring the Future project (Johnston et al., 2018) and assessed whether youth had used alcohol more than just a few sips, gotten drunk or buzzed from drinking alcohol, smoked cigarettes, or used tobacco, and smoked marijuana or used other illegal drugs during the past 30 days. Youth who indicated using at least one type of ATOD were coded as one, and youth who reported no use of any ATOD were coded as zero (Hamilton et al., 2009).

#### ***Fear of Deportation (***Cox et al., 2022***)***

Youth responses to a five-item Family Fear of Deportation Scale with a binary response set (yes, 1 and no, 0) assessed for different behavioral effects of FOD. The scale included items such as going without medical attention, limiting travel, and not participating in after-school activities due to fear of being deported. Items were summed with higher scores indicating more FOD. The reliability for the stress scale is KR20 = 0.62.

#### Stress

We assessed for stress using the eight-item PPSM (Bevans et al., 2018), validated for use with a Hispanic immigrant population (Sahbaz et al., 2022). The PPSM uses a five-point frequency scale (0, never to 4, always) and includes items such as “I felt overwhelmed” and “I felt unable to manage things in my life.” The reliability for the stress scale is *α* = 0.95.

#### Hope

Snyder’s Children’s Hope Scale (CHS; Snyder et al., 1997) was validated for use with Hispanic immigrant youth (Lin et al., 2022) and assessed the construct of hope using a 5-point Likert scale. The CHS (Lin et al., 2022) includes items such as “I can think of many ways to get the things in life that are most important to me,” and “When I have a problem, I can come up with lots of ways to solve it.” The reliability for the hope scale is *α* = 0.89.

#### Covariates and Auxiliary Variables

Gender (1, female and 0, male) is included in the analysis as a control variable. Demographic variables such as the duration of time a child has lived in the USA, the language the child used to respond to the survey (English vs. Spanish), parent marital status, father education, and mother education were also included in the analysis to identify potential confounders and to correct for missingness. All measures were assessed at all time points. For the current study, we drew FOD from Time 1 data, stress and hope from Time 2 data, and ATOD from Time 3 data.

### Missing Data and Plan of Analysis

Participants included two cohorts of first- and second-generation Latino youth (*N* = 126 for the first cohort and *N* = 74 for the second cohort). Whereas the youth in Cohort 1 completed a full version of the questionnaire at Time 1, Cohort-2 youth responded to a subset of the questionnaires under a planned-missing design (Graham, 2009; Graham et al., 2006; Little & Rubin, 2019; Palmer & Royall, 2010). All youth responded to demographic-related questions but were randomly assigned to answer one of six questionnaire forms. In the six-form planned-missing design (Table 1), stress was included in all six forms, and fear of deportation, hope, and ATOD were randomly assigned to two or three forms of the questionnaires.

**Table 1:** The six-form planed-missing design for cohort 2

	Form-1	Form-2	Form-3	Form-4	Form-5	Form-6
1. FOD-T1					X	X
2. Stress-T2	X	X	X	X	X	X
3. Hope-T2	X					X
4. ATOD-T3	X	X	X			
5. Female-T1	X	X	X	X	X	X

*FOD* fear of deportation, *ATOD* alcohol tobacco and other drug use, *T* data collection time point

With a planned-missing design, missingness is completely at random (MCAR) because the likelihood of missingness is not related to the variables of interest (Little & Rubin, 2019). Therefore, maximum likelihood methods or multiple imputation, which assumes missing completely at MCAR or missing at random (MAR), could be applied to handle missingness. Across both cohorts, the combined attrition rate from Time 1 to Time 2 was 20.5% (*N* = 159) and from Time 1 to Time 3 was 29.0% (*N* = 142). Table 2 shows the percent missing for each scale due to the combination of planned missingness and attrition. To handle missingness, sample characteristics such as child gender, the child’s duration of time living in the USA, the language the child used to respond to the survey, parent marital status, father education, and mother education were used to test their correlations with missingness (see Table 2). There were no significant sample characteristic differences between Cohort 1 and Cohort 2. Additionally, there were no significant differences on key variables (i.e., fear of deportation, stress, hope, and ATOD use) between the attrition cases and complete samples except for father’s education and child gender. The more educated fathers were more likely to quit the study at Time 2 than the less educated fathers. Males were also more likely to leave the study at Time 3 than females. Additionally, females were more likely to experience stress and less likely to have hope and use ATODs, compared to males. Preferred language to take the survey (i.e., English or Spanish) was correlated with FOD and father education. Thus, because gender was correlated with both the predictor variables and the outcome variable, we included it as a covariate in the model to control for its influence on the modeled associations. Because fathers’ education and preferred survey language were not correlated with mediators, moderators, or outcome variables, we included them only as auxiliary variables to enhance missing data computations and power (Graham, 2009; Little & Rubin, 2019).

**Table 2:** Intercorrelations for studied variables, demographic variables, and missingness.

	**1**	**2**	**3**	**4**	**5**	**6**	**7**	**8**	**9**	**10**	11
1. FOD-T1											
2. Stress-T2	.21*										
3. Hope-T2	-.08	-.35***									
4. ATOD-T3	.07	.33**	-.29**								
5. Female-T1	.02	.27**	-.20*	-.21*							
6. D-in-US-T1	.08	-.03	-.01	.08	.01						
7. Language used-T1	-.28**	.02	.01	-.11	.02	.01					
8. Marital status-T1	-.06	-.07	-.05	.04	-.03	-.08	.07				
9. Mother education-T1	0	.03	-.12	-.12	-.02	.01	-.02	-.06			
10. Father education -T1	-.02	-.05	.01	.13	.05	.10	.17*	.01	.68***		
11. Missingness (T1-2)	-.10	.04	.09	.16	-.02	.02	.03	.10	.11	.20**	
12. Missingness (T1-3)	-.15	-.06	.13	-.04	-.16*	.06	-.01	.09	.001	.06	.33***

FOD fear of deportation, ATOD alcohol tobacco and other drug use, D-in-US the duration of time the child was living in USA, T data collection time point. ^*^p < .05, ^**^p < .01, ^***^p < .00

**Fig. 2:**
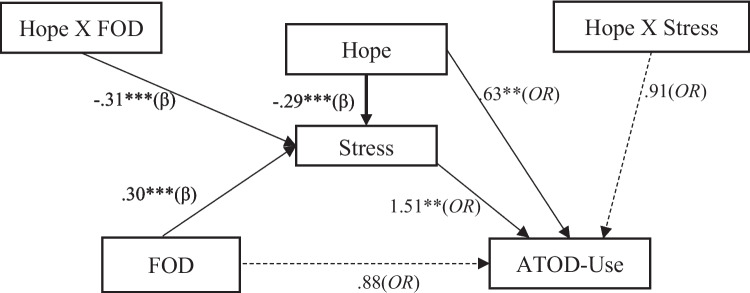
Hope moderated mediated path from fear of deportation to ATOD-use through stress, controlling for gender. Note: The area with diagonal lines indicates a significant region. The bold curved line is the regression line. The two thin curved lines are 95% interval bands. The vertical line represents the centered mean Hope score in the sample

We used Mplus 8.4 to model the hypothesized paths and conduct the moderated mediation model. We modeled stress as a mediator of the relationship between FOD and youth ATOD. In addition, hope was included as a moderator of the paths from FOD to stress and the path from stress to youth ATOD. Child gender was controlled for in all analyses (see Fig. 2). Due to the binary ATOD outcome, we used a weighted least squares mean and variance adjusted estimator (WLSMV; Suh, 2015). The WLSMV estimator was also used to handle missingness (Asparouhov & Muthén, 2010), which assumes missing at random (MAR). To make the MAR assumption more credible (Little, 1995), father education, child gender, and preferred survey language were used as auxiliary variables in the model (Little & Rubin, 2019). The scores of hope, stress, and FOD were grand-mean centered for a more direct reading of the results.

## Results

### Descriptive Statistics and Intercorrelations

The means, standard deviations, skewness, kurtosis, sample size, missingness, and correlation matrix are reported in Tables 2 and 3. Twenty-one percent of youth reported having used at least one of the substances included in the ATOD variable. FOD was not significantly correlated with ATOD (*r* = 0.07, *p* > 0.05). This does not support our first hypothesis. Stress at Time 2 was positively related with FOD at Time 1 (*r* = 0.21, *p* = 0.04), youth ATOD (*r* = 0.33, *p* < 0.001) at Time 3 and being female (*r* = 0.27, *p* < 0.001). Stress was inversely related with hope at Time 2 (*r* =  − 0.35, *p* < 0.001). Hope was inversely related with ATOD (*r* =  − 0.29, *p* = 0.004) and being female (*r* =  − 0.20, *p* = 0.03). ATOD was inversely related with being female (*r* =  − 0.21, *p* = 0.03).

**Table 3:** Means and standard deviations for study variables

	Mean	*SD*	*Skewness*	*Kurtosis*	*N*	Missing* (%)*
1. FOD-T1	1.57	1.21	.63	2.68	131	34.5
2. Stress-T2	1.97	1.05	.90	2.83	159	20.5
3. Hope-T2	3.40	.91	− .19	2.83	119	40.5
4. ATOD-T3	.21	.41	1.42	3.01	118	41.0
5. Female-T1	.49	.50	.05	1.00	200	0

*FOD* fear of deportation, *ATOD* alcohol tobacco and other drug use, *T* data collection time point

^*^p < .05, ^**^p < .01, ^***^p < .001

### Moderated Mediation Results

**Fig. 3:**
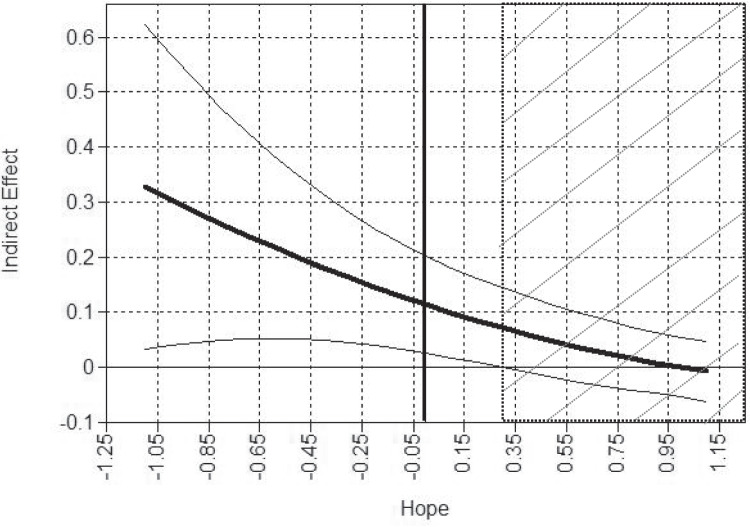
Hope moderates the indirect effect of fear of deportation on ATOD use through stress, using the Johnson-Newman technique

Global fit indexes showed an excellent model fit (i.e., *χ*^*2*^ = 12.61, *df* = 13, *p* = 0.53; *RMSEA* = 0.00, *CFI* = 1.00, *TLI* = 1.02, and *SRMR* = 0.05) for the moderated mediation model, and the model explained 52% of the variance in ATOD. The results for the moderated mediation model shown in Fig. 2 are as follows: (1) As we hypothesized, the path from FOD to youth ATOD was fully mediated through stress with significant paths from FOD to stress (*β* = 0.29, 95% CI [0.17, 0.40]) and from stress to ATOD (*OR* = 1.51, 95% CI [1.28, 1.78]). Furthermore, the direct path from FOD to youth ATOD was not significant (*OR* = 0.95, 95% CI [0.72, 1.25]); (2) hope was negatively associated with stress (*β* =  − 0.30, *p* < 0.001, 95% CI [− 0.43, − 0.16]) and moderated the path from FOD to stress (*β* =  − 0.31, 95% CI [− 0.42, − 0.20) but not the path from stress to ATOD (*OR* = 0.91, 95% CI [0.69, 1.19]). Using the Johnson-Neyman technique (Lin, 2020), Fig. 3 shows that the indirect effect of FOD on ATOD through stress depends on the value of hope. When hope increases, the indirect effect decreases. When hope is low (in the white area of Fig. 3), the indirect effect of FOD on greater ATOD through stress was significant. When hope was above the mean (in the area with diagonal lines of Fig. 3), the significant indirect effect disappeared. This suggests that FOD is associated with ATOD negatively through stress for Hispanic immigrant youth only when they are near or below average scores on hope.

A post-hoc power analysis for the model was run using R 4.2.2 to estimate the observed power of our study. Assuming an *α* of 0.05 and a sample size of 200, we have enough power (> 0.80) to detect the reported effects from FOD to stress, from hope to stress, from stress to ATOD use, from hope to ATOD, and the moderated effect from FOD to stress by hope. However, we were underpowered to detect the direct effect from FOD to ATOD (power = 0.06) and the moderated effect from stress to ATOD by hope (power = 0.05).

## Discussion

Due to the rapid population growth of Hispanic youth, the deleterious effects of early initiation of ATOD, and the unique stressors faced by Hispanic youth, it is imperative to continue the search for intervention targets that delay age of initiation and or reduce ATOD consumption among youth who have already begun use. In pursuit of this objective, we examined (a) the direct effects of FOD on ATOD, (b) whether this path was mediated by stress, and (c) if hope as defined by Gum & Snyder (2002) moderates the mediational model. Initial results indicate that FOD only predicts ATOD through its association with stress and that hope moderates the path from FOD to stress, but not from stress to ATOD. This suggests that hope is a potential target for interventions designed to reduce or delay ATOD among Hispanic youth. By increasing hope, the impact of FOD on stress is reduced. In fact, when hope is high (above the mean in the area with diagonal lines of Fig. 3), hope completely offsets the impact of FOD on our measure of stress. But if stress already exists, hope does not significantly influence the impact of stress on ATOD. Overall, the model suggests that hope influences the impact of FOD on youth ATOD by reducing the amount of stress related to deportation anxiety.

Although there is scant research from the field of adolescent substance use to explain how hope might offset the effects of the mediated path from FOD to ATOD through stress, other fields offer some initial insight. First, however, it is important to reiterate that hope is a dynamic combination of goal directed pathways and personal agency and that the concepts of agency and pathways are both necessary to the definition of hope; that is, neither is sufficient by itself. Similar concepts such as optimism, self-efficacy, and coping are related to hope yet are conceptually distinct. Optimism (Scheier & Carver, 1985) corresponds to Snyder’s pathways concept but lacks the notion of agency. On the other hand, self-efficacy (Bandura, 1985) corresponds to Snyder’s concept of agency but lacks the pathways concept. Coping has been defined by Lazarus and Folkman (1984, p. 141) as “constantly changing cognitive and behavioral efforts to manage specific external and/or internal demands that are appraised as taxing or exceeding the resources of the person.” Whether the individual appraises the demand as a challenge or a threat will lead to distinct types of coping (Lazarus & Folkman, 1984). For instance, when individuals appraise a stressor as a threat, they are more likely to respond with wishful thinking and support seeking. However, when they appraise the same stressor as a challenge, they are more likely to respond with goal-directed problem-focused coping (Folkman & Lazarus, 1985). According to Snyder, high-hoping individuals are more likely to view demands as challenging rather than as threatening (Snyder et al., 1991). This suggests that different levels of hope may prompt qualitatively distinct responses to stressors.

It may be that Hispanic adolescents with elevated levels of hope appraise the stress of deportation anxiety as a challenge rather than a threat and, therefore, are more efficient at finding alternative meaningful ways of addressing either their situation or their response to the situation through goal directed behaviors relative to their low-hope counterparts. Snyder (2002) proposes that when a goal is truly unobtainable, high-hoping individuals generate meaningful alternative goals and pathways that restore a sense of control or agency over the situation. The ability to generate and implement alternative goal-directed pathways, in turn, leads to effective positive coping and psychological well-being despite not being able to realize the original goal. This underscores that the essence of hope rests on the dynamic interplay between goal generated pathways and personal agency or perceived control to make a meaningful difference in one’s life trajectory despite untenable circumstances.

Although a meaningful change in immigration policy is likely not an attainable goal for immigrant Hispanic adolescents managing deportation anxiety, making minor changes to behavior may help to relieve stress by restoring a sense of control over their precarious situation. For these youth, the content of hope may be the negative goal of avoiding detection by the authorities and involve actionable, strategic behaviors (pathways) within their agency that promote this goal. Others have documented changes in behaviors designed to avoid detection including things like restricting travel, not seeking out medical attention, and participating in networks to communicate about potential raids and areas where authorities are actively stopping cars (Cox et al., 2022). While not fail-safe, these actions may provide youth with a purposeful focus and an increased sense of control sufficient to reduce their perceived stress.

Similarly, it may be that in generating pathways to help mitigate potential vulnerability, high-hope Hispanic adolescents are able to engender a sense of increased internal locus of control. Individuals whose locus of control is predominantly internal tend to feel more in control of their own lives and, therefore, focus their efforts on actionable coping strategies to manage current problems and experience less psychological distress (Rotter, 1966). Hope theory suggests that when a certain goal is blocked, high-hope individuals are flexible in changing their goals to ones that are more obtainable (Irving, 1998). That is, high personal agency fosters a sense of being able rather than simply being a victim of external forces. For instance, Papanikolaou et al., (2013) found that externality (i.e., external locus of control) was linked to increases in psychological distress independent of whether the source of the trauma was political conflict or other sources; in other words, it was the increasing sense of helplessness in controlling one’s life that produced the most extreme levels of distress. Others have also found inverse associations between internal locus of control and adolescent substance use (Dielman et al., 1987). Therefore, what seemingly reduces perceived stress is the extent individuals see themselves as able to generate pathways toward their goals, regardless of whether those pathways are successful.

Over the past half a century, a large body of empirical evidence has found that individuals often rely on alcohol and/or drugs to regulate the subjective experience of their internal affective states (e.g., Skrzynski, 2020). Our study adds to this research and suggests that as Hispanic immigrant youth perceive increased amounts of stress due to fearing that they or a significant other may be deported, they may increasingly turn to alcohol and drug use as a coping mechanism. However, to the degree that they have hope, that is, they are able to generate pathways in pursuit of their negative goal of avoiding being detected by the authorities and they perceive themselves as able to either implement these strategies or develop others, their perceived stress is reduced and, therefore, their need to use substances to cope.

The importance of this study’s findings for prevention efforts is fourfold. First, because hope is a malleable intervention target, it may be easily incorporated into existing prevention programs, which may aid in its dissemination. Second, training youth to be more hopeful does not focus on ATOD use. Many Hispanic immigrant families do not see substance use as a problem for their child and are reticent to attend substance use prevention programming. Programming themes and objectives that are aligned with family beliefs, values, and goals positively influence recruitment and retention, which in turn, may improve overall outcomes (Cox, Washburn, et al., 2021). Third, unlike most existing prevention interventions, teaching youth to be more hopeful does not require master’s level clinicians to deliver the content. Recent migratory trends show that Mexican heritage immigrants and immigrants from other non-English speaking countries are increasingly settling in new destination areas outside of traditional enclaves such as Texas, California, and Arizona. Because many of these new settlement areas do not have the social infrastructure necessary to attract bilingual service providers, prevention strategies that can be implemented with the available, local resources are urgently needed. Finally, in addition to fear of deportation, there are other potential factors which were not included in our study but may contribute to adolescent perceived stress (e.g., material hardship, discrimination). It will be important for future research to replicate the moderating effects of hope on the paths between different environmental stressors and adolescent perceived stress, to examine whether hope is a universal prevention strategy that has potential to influence the diverse child outcomes associated with psychosocial stress.

## Limitations

Despite the study’s many strengths, at least four limitations are worth noting. First, the study sample was limited to first- and second-generation Hispanic immigrant youth primarily from Mexican or Central American descent in the seventh grade. The extent individuals of Hispanic descent experience FOD varies by group membership (Moslimani, 2022). For example, second- and third-generation immigrants experience less fear than first-generation immigrants, and groups such as Puerto Ricans and Cubans who are either US citizens or eligible for the US Refugee Admissions Program experience less fear relative to people from other nationalities. Future research should diversify the sample to increase generalizability. Second, the measures were youth report, which exposes the study to a single source bias (Campbell & Fiske, 1959) and should be replicated using multiple sources where possible. Third, assessing for past 30-day ATOD at Time 3 may have missed youth who used a drug at Time 2 or Time 1 but not at Time 3. Future research may consider including ATOD at multiple timepoints to better capture the full range of substance using youth. Finally, our study was underpowered to reliably estimate the moderating effect of hope on the path from stress to ATOD use. Similarly, our study lacked power to determine the direct effect of FOD on past 30-day ATOD use. The power to detect the direct effect might have been influenced by our sample consisting of 7th graders, among whom only 21% reported using any of the substances included in the ATOD variable. Additionally, the binary nature of our outcome variable may also have influenced power. Future studies with larger sample sizes are needed to assess whether hope moderates the effect of stress on ATOD and the direct effect of FOD on ATOD.

## Conclusion

Approximately 5.5 million children under the age of 18 live in the USA with at least one unauthorized immigrant parent, of which 4.5 million are US citizens (Capps et al., 2016; United States Census Bureau, 2017); roughly 75% of immigrant children report experiencing fear that a loved one or they themselves may be deported (Gonzales et al., 2014; Gramlich & Scheller, 2021). The negative effects of chronic distress on mental and physical health are well established (Juster et al., 2010); therefore, to the extent that the fear of being deported creates a chronic state of heightened stress among immigrants, it would suggest a looming public health crisis accompanied by acute increases in ATOD as more and more individuals turn to substances to cope. Furthermore, due to of a lack of accessible, culturally appropriate prevention interventions for first- and second-generation Hispanic immigrants, heightened stress will almost undoubtedly result in even greater health disparities for this population.

Given the growing number of Hispanic immigrants in the USA and the likelihood of expanding health disparities facing this population, it is increasingly important to understand the social factors that affect their health. We believe this study is the first to quantitatively document the mediated path from fear of deportation to ATOD through perceived stress among Hispanic youth. Particularly encouraging is the ability of hope to moderate this path and interrupt the progression to ATOD among youth. Because hope is a malleable factor, it could be incorporated into a variety of prevention interventions. Thus, if these findings are replicated, promoting hope may prove to be one way to increase resilience and narrow the extant disparities in health among Hispanics, and in so doing, create a more equitable and inclusive society.
